# Situation, Education, Innovation, and Recommendation: A Large-Scale Systematic Review of Advance Care Planning in the Age of COVID-19

**DOI:** 10.3390/healthcare12060667

**Published:** 2024-03-15

**Authors:** Thomas Mayers, Ayaka Sakamoto, Ryota Inokuchi, Kyoko Hanari, Huijun Z. Ring, Nanako Tamiya

**Affiliations:** 1 Department of Health Services Research, Institute of Medicine, University of Tsukuba, Tsukuba 305-8575, Ibaraki, Japan; inokuchir-icu@md.tsukuba.ac.jp (R.I.); ntamiya@md.tsukuba.ac.jp (N.T.); 2 Medical English Communications Center, Institute of Medicine, University of Tsukuba, Tsukuba 305-8575, Ibaraki, Japan; 3 Department of Health Services Research, Graduate School of Comprehensive Human Sciences, University of Tsukuba, Tsukuba 305-8575, Ibaraki, Japan; ayaka.furukawa@gmail.com; 4 Health Services Research and Development Center, University of Tsukuba, Tsukuba 305-8575, Ibaraki, Japan; bewings@alto.ocn.ne.jp; 5 Hinohara Memorial Peace House Hospital, Nakai 259-0151, Kanagawa, Japan; 6 Department of Medicine, Stanford University, Stanford, CA 94305, USA

**Keywords:** advance care planning, COVID-19 pandemic, systematic review, barriers and facilitators, older adults

## Abstract

The COVID-19 pandemic highlighted the need for advance care planning (ACP) as a way to help mitigate the various care concerns that accompanied the healthcare crisis. However, unique obstacles to typical ACP practice necessitated the need for guidance and innovation to help facilitate these vital conversations. The aim of this systematic review was to identify the various ACP barriers and facilitators that arose during the pandemic and determine how ACP practice was affected across different contexts and among different populations. This systematic review (PROSPERO registration number: CRD42022359092), which adheres to the PRISMA guidelines for reporting systematic reviews, examined studies on ACP in the context of the COVID-19 pandemic. The review involved searches of five databases, including MEDLINE and Embase. Of the 843 identified studies, 115 met the inclusion criteria. The extracted ACP barriers and facilitators were codified and quantified. The most frequently occurring ACP barrier codes were: Social distancing measures and visitation restrictions, Uncertainty surrounding the COVID-19 prognosis, and Technological/Telehealth barriers. The most frequently occurring ACP facilitator codes were the following: Telehealth/virtual ACP platforms, Training for clinicians, and Care team collaboration. Identifying the ACP barriers and facilitators is essential for developing effective, resilient ACP promotion strategies and improving its delivery, accessibility, and acceptability.

## 1. Introduction

The COVID-19 pandemic was most devastating, in many respects, for older adults; not only was the SARS-Covid-2 virus more deadly in this population, but the damage caused by the social isolation that many faced was profound [1,2,3,4,5,6,7,8]. For older adults and other vulnerable populations, the pandemic brought to the forefront the critical importance of advance care planning (ACP). ACP is a well-established and evidenced practice that helps to ensure the wishes of a person, regarding their medical care and treatment towards the end of life, are respected should they become unable to communicate those preferences and complex healthcare decisions need to be made [9,10,11,12]. The unprecedented nature of the COVID-19 pandemic; however, characterized by rapid disease progression [13,14], healthcare system overload [15,16], and constantly evolving clinical guidelines [17,18], has posed significant challenges to ACP. Conversely, the pandemic has also potentially acted as a catalyst for enhancing the awareness and implementation of ACP due to the heightened perception of health risks [19,20,21].

The importance of ACP in aging societies, such as Japan, cannot be overstated [22,23]. As the demographic structure shifts towards a higher proportion of elderly individuals, the burden on healthcare systems intensifies, accompanied by an increase in chronic illnesses and end-of-life care needs [24,25]—a burden further exacerbated by the COVID-19 pandemic. ACP can aid in reducing this burden on healthcare systems by promoting effective resource utilization and preventing unnecessary or unwanted medical interventions [9,10,26]. Moreover, ACP can foster better communication and understanding between healthcare providers, patients, and their families, leading to more coordinated and compassionate care [27,28]. This alignment of care with patient preferences is especially important in preserving the dignity and quality of life of older adults. Therefore, the implementation of ACP can not only address the practical aspects of healthcare delivery in an aging society but can also resonate deeply with the cultural and ethical values surrounding care for older adults [22,29]. Thus, in 2022, after the immediate crisis of the pandemic had passed, in response to a request from the Japanese Ministry of Health, Labour, and Welfare, we sought to investigate how ACP practice had been impacted. Following our previously published umbrella review [30], the purpose of this current study was to explore the following questions: How did the COVID-19 pandemic affect the practice of ACP? What obstacles or barriers did the COVID-19 pandemic create to the delivery of ACP? What trends occurred to facilitate ACP practice during the pandemic?

To comprehensively explore these questions, our research employed the systematic review methodology, which allowed for a thorough and structured examination of the existing literature, capturing a wide array of experiences and findings from different healthcare settings and populations affected by the COVID-19 pandemic. By synthesizing data from multiple studies, we aimed to identify common themes and granular, divergent, or unique findings regarding the facilitators and barriers to ACP during this global health crisis. Identifying the factors that have either hindered or aided ACP during this period is not only essential for developing effective strategies to promote ACP in similar situations in the future, but also for improving ACP’s delivery, accessibility, and acceptability in general. The insights gained from this systematic review are expected to inform healthcare policy and practice, contributing to more resilient and patient-centered healthcare systems.

## 2. Materials and Methods

### 2.1. Protocol and Registration

The protocol for this systematic review was registered with the PROSPERO International Prospective Register of Systematic Reviews system (registration number: CRD42022359092; https://www.crd.york.ac.uk/prospero/display_record.php?ID=CRD42022359092, accessed on 15 November 2023). This systematic review strictly adheres to the Preferred Reporting Items for Systematic Reviews and Meta-Analyses (PRISMA) 2020 guidelines [31]. Covidence systematic review software (with Extraction version 2.0, Veritas Health Innovation, Melbourne, Australia), which is widely used for conducting systematic reviews and acknowledged by Cochrane as the foremost tool for initial screening and data extraction in standard intervention reviews [32], was used for all data management, including the study selection and data extraction processes [32].

### 2.2. Information Sources

Using the identified search strategies (Box 1), potentially relevant papers were identified from five databases: MEDLINE/PubMed, Embase, Cochrane Central Register of Controlled Trials, Web of Science, and Google Scholar. The strategy also included manually checking the reference lists of included studies to identify any further relevant studies. The search was restricted to the literature from peer-reviewed journals, including conference abstracts, published in 2020 or later. The search was performed by two researchers (T.M. and H.R.).

Box 1 **Search strategy used for each database.**

**1. Embase**

(‘covid-19’:ab OR ‘covid-19’:ti OR ‘covid-19’:kw OR ‘novel coronavirus disease’:ab OR ‘novel coronavirus disease’:ti OR ‘novel coronavirus disease’:kw OR ‘sars-cov-2’:ab OR ‘sars-cov-2’:ti OR ‘sars-cov-2’:kw OR ‘severe acute respiratory syndrome coronavirus 2’:ab OR ‘severe acute respiratory syndrome coronavirus 2’:ti OR ‘severe acute respiratory syndrome coronavirus 2’:kw) AND (‘advance care planning’:ab OR ‘advance care planning’:ti OR ‘advance care planning’:kw OR ‘advance care directive’:ab OR ‘advance care directive’:ti OR ‘advance care directive’:kw OR ‘living will’:ab OR ‘living will’:ti OR ‘living will’:kw) AND [2020–2022]/py

**2. MEDLINE/PubMed**

Search: (((((((COVID-19[Title/Abstract])) OR (novel coronavirus disease[Title/Abstract])) OR (SARS-CoV-2[Title/Abstract])) OR (severe acute respiratory syndrome coronavirus 2[Title/Abstract])) AND (advance care planning[Title/Abstract])) OR (advance care directive[Title/Abstract])) OR (living will[Title/Abstract]) Filters: Full text, from 2020 to 2022

**3. Web of Science**

((((((TS=(COVID-19)) OR TS=(“novel coronavirus disease”)) OR TS=(SARS-CoV-2)) OR TS=(“severe acute respiratory syndrome coronavirus 2”)) AND TS=(“advance care planning”)) OR TS=(“advance care directive”)) OR TS=(“living will”) 2020–2022

**4. Google Scholar**

allintitle: “COVID 19” OR “novel coronavirus disease” OR “SARS CoV 2” OR “severe acute respiratory syndrome coronavirus 2” AND “advance care planning” OR “advance care directive” OR “living will”

**5. Cochrane Library**

(“COVID 19” OR “novel coronavirus disease” OR “SARS CoV 2” OR “severe acute respiratory syndrome coronavirus 2”):ti,ab,kw AND (“advance care planning” OR “advance care directive” OR “living will”):ti,ab,kw

### 2.3. Study Selection

Covidence software was used for all aspects of the screening, data extraction, and quality assessment processes. Most duplicates were automatically removed by the Covidence software at the point of importing the studies; however, during the later screening process, some duplicate studies were further identified and removed manually. The titles and abstracts of the remaining citations were individually screened by two researchers (T.M. and A.S.). Irrelevant texts were excluded based on the following exclusion criteria: (1) not mentioning ACP (or other synonymous terms); (2) not mentioning the COVID-19 pandemic; (3) giving no indication of facilitators of or barriers to ACP; and (4) other reasons such as wrong setting, study design, etc. Review articles were excluded from the study but were used for manual citation searching for further relevant studies. Conflicting votes were resolved through discussion, before moving onto review of the full texts. Full text review was performed in the same manner by the same two researchers (T.M. and A.S.). An inter-rater reliability assessment was performed and extracted using the Covidence software.

### 2.4. Data Extraction

The data extraction form was created within the Covidence software. Data extraction was conducted according to the study characteristics. The extraction form contained the following items: study identification (ID), title, lead author, publication year, country in which the study was conducted, publication category, aim of study, study design, setting, population description, total number of participants, barriers, facilitators, increase or decrease in ACP during COVID-19 (Yes/No, value), and a summary of the main findings and implications/recommendations. Before beginning the data extraction process, the extraction form was tested on a number of studies and refined. During this process, four key categories emerged: (1) Situation—studies that described the situation regarding ACP practice; (2) Education—studies that described public- or professional-facing ACP-related educational programs/interventions; (3) Innovation—studies that described new ACP-related innovations; and (4) Recommendation—documents from experts or organizations that gave recommendations on ACP practice. Because of the large number of included studies, the data extraction process and quality assessment were conducted primarily by one researcher (T.M.) and were reviewed by another (A.S.), while collaborators oversaw all the data extraction processes. Data were exported from Covidence and presented as tables. The extracted data pertaining to the ACP barriers and facilitators were coded using a simple labeling system in which codes were assigned to categorize and quantify each barrier and facilitator.

### 2.5. Quality Appraisal

Quality assessment tools were created within the Covidence software. The Mixed Methods Appraisal Tool (MMAT) [33] was used for the assessment of qualitative and quantitative studies. Appraisal of nonrandomized trials assessed the following criteria: participant selection, outcome measures, outcome data, confounding factors, and intervention administration. Appraisal of quantitative descriptive studies assessed the following criteria: sampling strategy, study population, appropriateness of measures, nonresponse bias risk, and statistical analysis. Appraisal of randomized controlled trials assessed the following criteria: randomization methodology, group allocation, outcome data, blinding, and intervention adherence. For qualitative studies, assessment was made of the following criteria: methodology, data collection methods, derivation of the findings, data interpretation, and coherence between the data source, collection analysis, and interpretation. For studies that fell outside of the scope of MMAT assessment, such as letters, recommendations, reports, and guidelines, the AACODS checklist was used [34]. This tool, developed for appraisal of gray literature, gives the appraiser various questions to consider for probing and evaluating the authority, accuracy, coverage, objectivity, date, and significance of a study. Quality assessment data was exported from Covidence, synthesized, and presented visually in the tables using the following color-coded labeling system: red = low; orange = low-middle; yellow = high-middle; and green = high. Quality assessment data were not used to exclude studies.

## 3. Results

### 3.1. Overview of the Results

**Figure 1:**
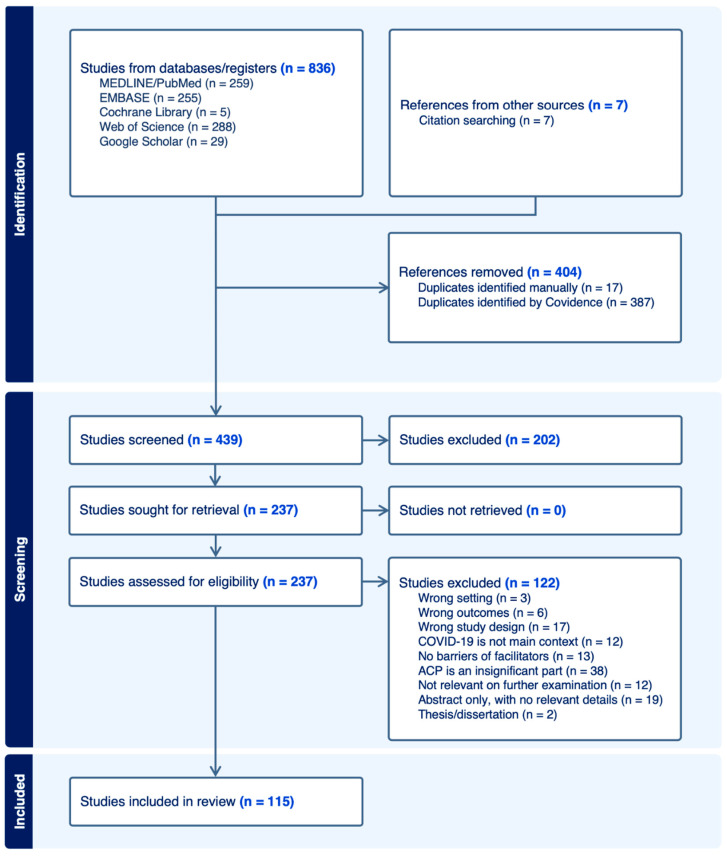
PRISMA diagram showing flow of search screening, exclusion, and inclusion of studies for the current review.

The search of the five databases yielded a total of 836 studies, and a search of citations yielded a further 7 studies. The Covidence software automatically removed 387 duplicate studies, and a further 17 were removed manually. Of the remaining 439 studies, 202 irrelevant studies were excluded after screening the titles and abstracts. The remaining 237 studies underwent full-text screening and were assessed for eligibility and a further 122 studies were excluded. Finally, 115 studies that met the inclusion criteria were included in the review. Cohen’s Kappa values of 0.557 and 0.559 for the title/abstract and full-text screening showed moderate agreement between the two reviewers. The PRISMA flowchart detailing the study selection process is presented in Figure 1.

Table 1 shows the characteristics of the included studies. These comprised 34 (29.6%) nonrandomized studies, 28 (24.3%) letters/opinions/editorials, 20 (17.4%) quantitative descriptive studies, 19 (16.3%) qualitative studies, 7 (6.1%) reports (including case reports), and 7 (6.1%) were of mixed methods. Of the 115 included studies, 31 (27.0%) were conference abstracts. The included studies came from 13 different counties (in alphabetical order): Argentina (*n* = 1, 0.9%), Australia (*n* = 1, 0.9%), Belgium (*n* = 2, 1.7%), Canada (*n* = 6, 5.2%), Ireland (*n* = 1, 0.9%), Italy (*n* = 2, 1.7%), Japan (*n* = 1, 0.9%), Portugal (*n* = 1, 0.9%), Spain (*n* = 1, 0.9%), Taiwan (*n* = 2, 1.7%), Thailand (*n* = 1, 0.9%), the Netherlands (*n* = 5, 4.3%), the United Kingdom (*n* = 21, 18.3%), and the United States (*n* = 70, 60.9%). A range of different settings were represented among the studies, with the most frequent being hospitals (*n* = 42, 36.5%; including clinics, emergency departments, etc.), nursing homes (*n* = 21, 18.3%; including long-term care facilities, residential homes, etc.), and online/telehealth (*n* = 15, 13.0%). Study participants were largely patient populations of various kinds (*n* = 58, 50.4%; including those with COVID-19, inpatients, outpatients, nursing home residents, diagnosed with dementia, etc.) and healthcare professionals (*n* = 34, 29.6%; including doctors, nurses, residents, nursing home staff, etc.).

### 3.2. ACP Barriers and Facilitators during the COVID-19 Pandemic

Table 2, Table 3, Table 4 and Table 5 show the included studies divided into the above-mentioned four categories as follows: Situation (*n* = 35; Table 2), Education (*n* = 22; Table 3), Innovation (*n* = 32; Table 4), and Recommendation (*n* = 26; Table 5). The tables focus on the barriers to and facilitators of ACP during the pandemic identified in each of the studies.

#### 3.2.1. Situation Category

The 35 studies included in the Situation category are presented in Table 2. These studies primarily describe the situation of ACP practice during the COVID-19 pandemic across different settings and populations. Included in the Table is the Study ID (comprised of the first author’s surname and year of publication), country in which the study was conducted, study setting (hospital, nursing home, etc.) and population, number of participants (when available), quality assessment (color-coded system), and the coded barriers and facilitators. Further details for each study included in this category are available in Table S1.

**Table 2:** ACP barriers and facilitators during the COVID-19 pandemic: Situation category.

Study ID	Country	Setting	Population	Number of Participants	Quality Assessment	ACP Barriers and Facilitators
Mota Romero 2022 [35]	SP	NH	HP	20	■	Barriers: Lack of awareness/knowledge of ACP
Statler 2022 [36]	US	HO	PT	356	■	Barriers: Legal concerns; Uncertainty surrounding the COVID-19 prognosis; Limited resources; Healthcare system barriers; Discomfort among clinicians and patients discussing end-of-life care Facilitators: Resources/education for patients/families; Proactive ACP conversations; Telehealth/virtual ACP platforms; Identification of those in need of ACP
Porteny 2022 [37]	US	HO	PT	76	■	Barriers: Social distancing measures and visitation restrictions Facilitators: Telehealth/virtual ACP platforms
Epler 2022 [38]	US	HO	PT	720	■	Barriers: Discomfort among clinicians and patients discussing end-of-life care; Familial disagreement; Time constraints; Rapid disease progression
Kaehr 2022 * [39]	US	NH	HP	17	■	Barriers: Personal protective equipment requirements; Social distancing measures and visitation restrictions; Uncertainty surrounding the COVID-19 prognosis
Sun 2022 [40]	US	HO	PT	276	■	Barriers: Social distancing measures and visitation restrictions; Personal protective equipment requirements; Strained healthcare system; Uncertainty surrounding the COVID-19 prognosis Facilitators: Identification of those in need of ACP
Barnato 2022 [41]	US	ED	PT	5394	■	Facilitators: Diagnosis of dementia
Ter Brugge 2022 [42]	NL	NH	HP	127	■	Barriers: Social distancing measures and visitation restrictions Facilitators: Understanding/fear of COVID-19; Telehealth/virtual ACP platforms; Public awareness of ACP
Perumalswami 2022 [43]	US	HO	HP	22	■	Barriers: Technological/telehealth barriers
Jayes 2022 [44]	UK	HO, NH	HP	107	■	Barriers: Social distancing measures and visitation restrictions; Personal protective equipment requirements; Time constraints
Janssen 2021 [45]	NL	HO	PT		■	Barriers: Uncertainty surrounding the COVID-19 prognosis; Time constraints; Negative perceptions about advance care planning Facilitators: Public awareness of ACP; Telehealth/virtual ACP platforms; Training for clinicians; Guidance and protocols for ACP discussions; Care team collaboration
Brophy 2021 [46]	US	SV	PB	522	■	Barriers: Emotional barriers Facilitators: Understanding/fear of COVID-19
Piers 2021 [47]	BL	HO	PT	711	■	Facilitators: ACP/palliative care experts: co-management by geriatricians
Bradshaw 2021 [48]	UK	NH	HP	277	■	Barriers: Social distancing measures and visitation restrictions; Personal protective equipment requirements; Strained healthcare system; Rapid disease progression; Emotional barriers; Uncertainty surrounding the COVID-19 prognosis Facilitators: Innovation and flexibility in ACP documentation processes; Public awareness of ACP; Telehealth/virtual ACP platforms; Care team collaboration; ACP/palliative care experts; Training for clinicians
Dujardin 2021 [49]	NL	HO	HP	15	■	Barriers: Uncertainty surrounding the COVID-19 prognosis; Discomfort among clinicians and patients discussing end-of-life care; Technological/telehealth barriers; Social distancing measures and visitation restrictions; Time constraints; Healthcare system barriers Facilitators: Trusting clinical relationship; Telehealth/virtual ACP platforms; Healthcare system improvements
Connellan 2021 [50]	IR	ED	PT	430	■	Facilitators: Understanding/fear of COVID-19
Vellani 2021 [51]	CN	NH	HP	14	■	Facilitators: ACP/palliative care experts; Telehealth/virtual ACP platforms; Care team collaboration; Proactive ACP Conversations
Toccafondi 2021 * [52]	IT	HO	PT	110	■	Facilitators: Guidance and protocols for ACP discussions
DeGette 2021 * [53]	US	HO	PT	258	■	Barriers: Personal protective equipment requirements; Social distancing measures and visitation restrictions; Uncertainty surrounding the COVID-19 prognosis; Racial and ethnic barriers Facilitators: Identification of those in need of ACP
Copley 2021 [54]	UK	HO	PT	164	■	Facilitators: Resources for clinicians; Understanding/fear of COVID-19; Public awareness of ACP; Resources/education for patients/families
Lin 2021 [55]	TW	HO	PT	2493	■	Barriers: Social distancing measures and visitation restrictions; Lack of awareness/knowledge of ACP; Healthcare system barriers; Limited resources; Legal concerns; Discomfort among clinician and patients discussing end-of-life care; Time constraints; Strained healthcare system Facilitators: Telehealth/virtual ACP platforms
Ye 2021 [56]	US	NH	PT	963	■	Barriers: Social distancing measures and visitation restrictions Facilitators: Proactive ACP conversations
Huayanay 2021 [57]	US	HO	PT	1	■	Barriers: Communication difficulties; Cultural and religious beliefs; Financial concerns
Nguyen 2021 [58]	US	SV	PT	100	■	Facilitators: Public awareness of ACP; Improved messaging
Coles 2020 * [59]	UK	NH	HP		■	Facilitators: Care team collaboration; ACP/palliative care experts; Healthcare system improvements
Wei 2020 [60]	US	HO	HP		■	Barriers: Rapid disease progression; Social distancing measures and visitation restrictions Facilitators: Telehealth/virtual ACP platforms; Tablet computers; ACP/palliative care experts
Maia 2020 * [61]	PO	HO	PT	51	■	Barriers: Personal protective equipment requirements; Social distancing measures and visitation restrictions; Strained healthcare system; Technological/telehealth barriers
Hendriks 2022 [62]	NL	HO	PT	275	■	Facilitators: Training for clinicians; Resources for clinicians
Holdsworth L.M. 2022 [63]	US	HO	HP	15	■	Barriers: Technological/telehealth barriers; Social distancing measures and visitation restrictions Facilitators: Innovation and flexibility in ACP documentation processes
Dassel 2021 [64]	US	HC	FC	82	■	Barriers: Emotional barriers; Social distancing measures and visitation restrictions; Lack of awareness/knowledge of ACP Facilitators: Guidance and protocols for ACP discussions; Resources/education for patients/families
de Vries 2021 [65]	CN	SV	PB	3923	■	Barriers: Distrust in the healthcare system Facilitators: Resources/education for patients/families
Payne 2022 [66]	US	ED	PB	50	■	Barriers: Lack of awareness/knowledge of ACP Facilitators: Innovation and flexibility in ACP documentation processes; Resources/education for patients/families; Improved messaging
Ninteau 2022 [67]	US	NH	HP	7	■	Barriers: Social distancing measures and visitation restrictions Facilitators: ACP/palliative care experts; Training for clinicians; Telehealth/virtual ACP platforms
Elizondo 2022 * [68]	AR	HO	PT		■	Facilitators: Care team collaboration; Innovation and flexibility in ACP documentation processes; Healthcare system improvements
Hafid 2022 * [69]	CN	HO	HP	48	■	Facilitators: Public awareness of ACP; Resources/education for patients/families; Telehealth/virtual ACP platforms

Notes: * = Conference abstract. Abbreviations: Country: SP = Spain; US = The United States; NL = The Netherlands; UK = United Kingdom; BL = Belgium; IR = Ireland; CN = Canada; IT = Italy; TW = Taiwan; PO = Portugal; AR = Argentina. Setting: AC = Academic; HO = Hospitals; NH = Nursing homes; OT = Online/Telehealth; ED = Electronic Data; SV = Survey. Population: HP = Healthcare professionals; PT = Patients; PB = General Public; FC = Family Caregivers. Quality Assessment (level of evidence): ■ = Low; ■ = Low-Medium; ■ = High-Medium; ■ = High.

Across the included studies within this “situation” category, one consistent theme was how the pandemic highlighted the need for improved communication and awareness among healthcare professionals, patients, and their families with regards to ACP. For example, the NUHELP program’s findings [35] and observations from nursing homes [39], healthcare providers [48], and general practitioners [49] all point to the need for ACP discussions and familiarity with ACP processes. A study by Porteny et al., described how clinicians perceived an increased patient willingness to discuss quality of life and ACP due to COVID-19, but patients reported minimal engagement in such discussions [37]. This discrepancy was further exacerbated by challenges such as PPE hindering communication [44], remote discussions [42], and disparities in ACP knowledge across different communities [57].

A study by Statler et al. found that documentation of ACP, including code status and end-of-life preferences, was notably low (22.8%) for hospitalized COVID-19 patients [36]. However, palliative care consultations showed a positive correlation with ACP documentation [36,40], indicating the role of such consultations in the ACP process. The pandemic spurred an earlier initiation of ACP conversations, often influenced by media coverage and public awareness [45], but the quality of these discussions varied, with telemedicine emerging as a key but imperfect tool [43,44]. The variability in treatment intensity for patients with dementia [41] and the challenges in involving patients in shared decision-making [62] suggest that provider biases and systemic issues in healthcare communication persist.

The response to these issues has been multifaceted. Some institutions have seen an increase in the use of telemedicine for ACP [69], while others have emphasized the importance of personalized ACP [59] and integrated palliative care [51]. Redeployment of specialists [60] and creative solutions to facilitate discussions and legal documentation [63] have been implemented to address the challenges posed by the pandemic. This underscores the need for ongoing education and a more compassionate approach to encourage ACP discussions [66], not just during crises but as a standard practice in healthcare.

#### 3.2.2. Education Category

Table 3 shows the 22 studies included in the Education category (Table S2 gives further details on each study). These studies have a strong focus on ACP-related educational interventions that were aimed at practicing healthcare professionals, medical students, and patients and their family members. 

**Table 3:** ACP barriers and facilitators during the COVID-19 pandemic: Education category.

Study ID	Country	Setting	Population	No. of Participants	Quality Assessment	ACP Barriers and Facilitators
van de Wiel 2022 [70]	BL	AC	MS	172	■	Facilitators: Training for clinicians
Casey 2022 [71]	US	HO	PT	143	■	Facilitators: Training for clinicians; Guidance and protocols for ACP discussions; Innovation and flexibility in ACP documentation processes
Cousins 2022 [72]	UK	NH	HP, FM	54	■	Facilitators: Resources/education for patients/families; Resources for clinicians; Training for clinicians
Rosedale 2022 * [73]	US	HO	PT		■	Facilitators: Training for clinicians; ACP/palliative care experts
Rabow 2021 [74]	US	OT	HP		■	Facilitators: Training for clinicians; Resources for clinicians
Oulton 2021 [75]	US	HO	HP	9	■	Facilitators: Training for clinicians
Budidi 2021 * [76]	US	AC	HP	30	■	Barriers: Time constraints; Low education level; Communication difficulties; Technological/telehealth barriers; Lack of adequate ACP training for clinicians Facilitators: Training for clinicians
Price 2021 * [77]	US	HO	PT	143	■	Facilitators: Training for clinicians
Roberts 2020 [78]	US	AC	HP		■	Facilitators: Training for clinicians
Markwalter 2022 * [79]	US	HO	PT	143	■	Facilitators: Training for clinicians; Resources for clinicians
Preston 2022 * [80]	UK	NH	HP, NH, FM		■	Barriers: Technological/telehealth barriers Facilitators: Training for clinicians; Resources/education for patients/families
Cooney 2022 [81]	US	AC	MS	83	■	Facilitators: Training for clinicians
Holdsworth 2022 * [82]	UK	HO	PT	69	■	Facilitators: Training for clinicians; Identification of those in need of ACP; Guidance and protocols for ACP discussions
Volandes 2022 [83]	US	HO	PT	14107	■	Facilitators: Resources/education for patients/families; Training for clinicians
Mills 2021 [84]	US	HO	HP	48	■	Facilitators: Telehealth/virtual ACP platforms
Varey 2021 * [85]	UK	OT	HP, PT, FM		■	Facilitators: Resources/education for patients/families
Phenwan 2021 [86]	TL	OT	MS, HP, PB	103	■	Barriers: Cultural and religious beliefs; Lack of awareness/knowledge of ACP Facilitators: Training for clinicians
Berning 2021 [87]	US	NH	PT	581	■	Facilitators: Care team collaboration; Identification of those in need of ACP; Guidance and protocols for ACP discussions
Dobert 2021 * [88]	US	NH	HP		■	Facilitators: Care team collaboration; Training for clinicians; Improved messaging; Resources for clinicians; Telehealth/virtual ACP platforms
Smith 2020 [89]	US	OT	PT	413	■	Facilitators: Training for clinicians
McAfee 2022 [90]	US	AC	PB, HP		■	Barriers: Cultural and religious beliefs; Racial and ethnic barriers; Healthcare system barriers; Lack of awareness/knowledge of ACP; Low health literacy; Limited resources: accessibility to health directives
Huang 2021 [91]	US	HO	HP		■	Barriers: Discomfort among clinicians and patients discussing end-of-life care; Lack of adequate ACP training for clinicians Facilitators: Improved messaging

Notes: * = Conference abstract. Abbreviations. Country: US = The United States; UK = United Kingdom; BL = Belgium; TL = Thailand; Setting: AC = Academic; HO = Hospitals; NH = Nursing homes; OT = Online/Telehealth; ED = Electronic Data; SV = Survey. Population: HP = Healthcare professionals; PT = Patients; FM = Family; MS = Medical students; PB = General Public. Quality Assessment (level of evidence): ■ = Low; ■ = Low-Medium; ■ = High-Medium; ■ = High.

Education interventions for ACP during the COVID-19 pandemic have proven crucial for both the public and healthcare professionals, as evidenced by a variety of studies within the “education” category [70,71,72,73,74,75]. Training programs have shown a positive impact on the confidence of healthcare providers and students in initiating ACP discussions and recognizing patient cues, despite the challenges presented by social distancing and the need for remote communication [72,84]. This training has led to revised ACP procedures and empowered families to partake in care decisions [80], highlighting the importance of ACP as an ongoing, iterative process that is highly individualized and extends beyond medical and end-of-life considerations [86].

Despite the reported advancements, disparities persist, with a higher percentage of white patients meeting ACP quality standards post-intervention compared to non-white patients [73]. One study suggested that the pandemic has underscored the need to integrate death education more broadly into teacher training and undergraduate curricula to address cultural taboos and educate the public on ACP [90]. Virtual interventions, like online workshops and telemedicine curricula, have broadened the reach and accessibility of ACP education, proving to be effective in engaging diverse audiences [89,91]. Narrative-based education [78], virtual training sessions [75], and electronic resources [85] have emerged as valuable methods to facilitate ACP-related decisions and discussions. Overall, these studies indicate that while the pandemic posed challenges, it also provided an opportunity to enhance the understanding and implementation of ACP through public- and professional-facing educational interventions, making it more accessible and relevant for a wider population.

#### 3.2.3. Innovation Category

The Innovation category included 32 studies, which are presented in Table 4 (further details are giving in Table S3). These studies describe innovations that aided ACP practice and delivery during the pandemic. 

**Table 4:** ACP barriers and facilitators during the COVID-19 pandemic: Innovation category.

Study ID	Country	Setting	Population	No. of Participants	Quality Assessment	ACP Barriers and Facilitators
Finger 2022 [92]	US	NH	PT	24	■	Barriers: Social distancing measures and visitation restrictions; Strained healthcare system Facilitators: Care team collaboration; Guidance and protocols for ACP discussions
Hannon 2022 * [93]	CN	NH, HO	PT	26	■	Facilitators: Telehealth/virtual ACP platforms
Hui 2022 * [94]	US	HO	PT	12,941	■	Facilitators: Guidance and protocols for ACP discussions; Identification of those in need of ACP; Care team collaboration; Training for clinicians; Healthcare system improvements
Zhukovsky 2022 * [95]	US	HO	PT	76	■	Facilitators: Care team collaboration
Gessling 2022 * [96]	US	OT	PT	294	■	Facilitators: Telehealth/virtual ACP platforms; Care team collaboration
Vellani 2022 [97]	CN	HO	PT	21 dyads	■	Barriers: Emotional barriers Facilitators: ACP/palliative care experts; Training for clinicians; Public awareness of ACP; Resources/education for patients/families
Yen 2022 [98]	TW	HO	PT	897	■	Barriers: Uncertainty surrounding the COVID-19 prognosis; Limited resources Facilitators: Identification of those in need of ACP
Liberman 2022 [99]	US	HO	PT	64	■	Facilitators: Telehealth/virtual ACP platforms; ACP/palliative care experts
Meyers 2022 * [100]	US	OT	VT	106	■	Barriers: Social distancing measures and visitation restrictions Facilitators: Identification of those in need of ACP; Telehealth/virtual ACP platforms
Yourman 2022 * [101]	US	HO	PT	53	■	Facilitators: Training for clinicians; Telehealth/virtual ACP platforms
MacInnes 2022 * [102]	UK	OT	HP		■	Facilitators: Telehealth/virtual ACP platforms
Allen 2021 * [103]	UK	OT	PT, HP, PB		■	Facilitators: Resources for clinicians
Acevedo Rodriguez 2021 * [104]	US	OT	VT	500	■	Facilitators: Telehealth/virtual ACP platforms
Singh 2021 [105]	US	HO	PT		■	Facilitators: Care team collaboration; Healthcare system improvements
Nandhra 2021 * [106]	UK	NH	PT	585	■	Facilitators: Identification of those in need of ACP; ACP/palliative care experts
Paladino 2021 [107]	US	HO	HP		■	Facilitators: Guidance and protocols for ACP discussions
Lin 2020 [108]	US	ED	HP		■	Barriers: Social distancing measures and visitation restrictions Facilitators: Telehealth/virtual ACP platforms; Healthcare system improvements; Tablet computers; Language support services; Training for clinicians
Handalage 2020 * [109]	UK	OT	PT	160	■	Facilitators: Resources for clinicians; ACP/palliative care experts
Schoenherr 2020 [110]	US	HO	PT	29	■	Barriers: Social distancing measures and visitation restrictions Facilitators: Identification of those in need of ACP
Langmaid 2020 [111]	US	OT	PT		■	Facilitators: Care team collaboration; Telehealth/virtual ACP platforms
Gaur 2020 [112]	US	NH	HP	10	■	Barriers: Social distancing measures and visitation restrictions Facilitators: Guidance and protocols for ACP discussions
Bhatia 2021 [113]	US	HO	PT	356	■	Barriers: Social distancing measures and visitation restrictions Facilitators: Telehealth/virtual ACP platforms; Proactive ACP conversations
Cavalier 2020 [114]	US	HO	PT		■	Facilitators: Innovation and flexibility and in ACP documentation processes
Janssen 2020 [115]	NL	SV	HP	68	■	Facilitators: Proactive ACP conversations
Baharlou 2020 [116]	US	OT	PT	183	■	Facilitators: Innovation and flexibility in ACP documentation processes; Understanding/fear of COVID-19; Healthcare system improvements; Care team collaboration; Training for clinicians; ACP/palliative care experts; Telehealth/virtual ACP platforms
McFarlane 2022 * [117]	UK	ED	PB	56,343	■	Facilitators: Innovation and flexibility in ACP documentation processes
Mulyak 2021 * [118]	UK	ED	PT	107,614	■	Facilitators: Innovation and flexibility in ACP documentation processes
Hurlow 2021 [119]	UK	ED	PT		■	Facilitators: Innovation and flexibility in ACP documentation processes
Funk 2020 [120]	US	ED	PB		■	Facilitators: Telehealth/virtual ACP platforms; Innovation and flexibility in ACP documentation processes
Portz 2020 [121]	US	OT	PB	3292	■	Facilitators: Innovation and flexibility in ACP documentation processes; Resources/education for patients/families
Schifeling 2020 [122]	US	OT	PT	190	■	Facilitators: Telehealth/virtual ACP platforms
Auriemma 2020 [123]	US	OT	PT, FM		■	Facilitators: Resources/education for patients/families

Notes: * = Conference abstract. Abbreviations. Country: US = The United States; NL = The Netherlands; UK = United Kingdom; CN = Canada; TW = Taiwan; Setting: AC = Academic; HO = Hospitals; NH = Nursing homes; OT = Online/Telehealth; ED = Electronic Data; SV = Survey. Population: HP = Healthcare professionals; PT = Patients; FM = Family; PB = General Public; VT = Veterans. Quality Assessment (level of evidence): ■ = Low; ■ = Low-Medium; ■ = High-Medium; ■ = High.

The COVID-19 pandemic served as a catalyst for various innovations that led to increases in the engagement of patients in ACP [104,116,123]. The adoption of telehealth technologies and electronic platforms, such as eMOLST, facilitated this increase, allowing patients and families to discuss ACP remotely and ensure their care preferences are documented and respected [96,109]. Due to visitation restrictions, patients and healthcare providers employed virtual tools for ACP discussions, leading to significant improvements in ACP documentation [99,102,117].

Initiatives like the BRIDGES program [111] and telemedicine curricula [97] further broadened the scope and accessibility of ACP discussions, ensuring that even during the pandemic, care remained aligned with patient values and goals. Meanwhile, healthcare providers saw the importance of proactive ACP in reducing unwanted interventions and healthcare costs [113] and made strides towards incorporating ACP into standard practice, as evidenced by the integration of ACP into electronic health records and the widespread use of care planning tools [108,118]. Furthermore, one study demonstrated that ACP led to cost benefits for healthcare systems and enhanced patient quality of life, especially in frail populations [106].

Despite the complexity of initiating ACP discussions and the preference for in-person encounters, some studies demonstrated that meaningful ACP conversations can occur remotely, with tailored approaches to individual needs [100,101]. This shift was also reflected in the increased utilization of patient portals for ACP [121], and the expansion of ACP discussions to address a wider range of medical issues during telehealth visits [122]. The pandemic’s pressures underscored the need for ACP to be routine, adaptable, and patient-centered [107,110,115], and, moving forward, the innovations made during the pandemic were believed to help facilitate those goals.

#### 3.2.4. Recommendation Category

The 26 manuscripts included in the Recommendation category are shown in Table 5 (extra details are presented in Table S4). The works included here represent documents from experts in the field of ACP or organizations that gave recommendations concerning ACP practice at a time of great upheaval in the healthcare system. For this category, the table includes only the Study ID, country, quality assessment, and coded barriers and facilitators.

**Table 5:** ACP barriers and facilitators during the COVID-19 pandemic: Recommendation category.

Study ID	Country	Quality Assessment	ACP Barriers and Facilitators
Auriemma 2022 [124]	US	■	Barriers: Social distancing measures and visitation restrictions; Technological/telehealth barriers; Uncertainty surrounding the COVID-19 prognosis; Negative perceptions about advance care planning; Distrust in the healthcare system; Racial and ethnic barriers Facilitators: Identification of those in need of ACP; Guidance and protocols for ACP discussions; ACP/palliative care experts
Back 2021 [125]	US	■	Barriers: Lack of awareness/knowledge of ACP; Uncertainty surrounding the COVID-19 prognosis; Distrust in the healthcare system Facilitators: Improved messaging
Bender 2021 [126]	US	■	Barriers: Time constraints; Limited resources; Healthcare system barriers; Discomfort among clinicians and patients discussing end-of-life care; Strained healthcare system; Personal protective equipment requirements Facilitators: Telehealth/virtual ACP platforms; Care team collaboration; Innovation and flexibility in ACP documentation processes
Block 2020 [127]	US	■	Barriers: Legal concerns; Social distancing measures and visitation restrictions Facilitators: Care team collaboration; Innovation and flexibility in ACP documentation processes; Resources/education for patients/families; Telehealth/virtual ACP platforms
Chan 2020 [128]	UK	■	Barriers: Limited resources; Familial disagreement; Uncertainty surrounding the COVID-19 prognosis; Lack of awareness/knowledge of ACP; Social distancing measures and visitation restrictions Facilitators: Telehealth/virtual ACP platforms; Innovation and flexibility in ACP documentation processes
Chase 2020 [129]	CN	■	Facilitators: Telehealth/virtual ACP platforms; Healthcare system improvements; Guidance and protocols for ACP discussions
Curtis 2020 [130]	US	■	Barriers: Strained healthcare system Facilitators: Telehealth/virtual ACP platforms; Care team collaboration
Dattolo 2021 [131]	IT	■	Barriers: Social distancing measures and visitation restrictions Facilitators: Telehealth/virtual ACP platforms
Dewhurst 2021 [132]	UK	■	Facilitators: Proactive ACP Conversations
Farrell 2020 [133]	US	■	Facilitators: Healthcare system improvements; Care team collaboration: teamwork/multi-disciplinary involvement; Telehealth/virtual ACP platforms; Identification of those in need of ACP
Gordon 2020 [134]	UK	■	Barriers: Social distancing measures and visitation restrictions; Blanket approaches to ACP; Negative perceptions about advance care planning Facilitators: ACP/palliative care experts; Individualized ACP discussion
Hill 2021 [135]	UK	■	Barriers: Social distancing measures and visitation restrictions; Personal protective equipment requirements Facilitators: Telehealth/virtual ACP platforms; Early ACP; Trusting clinical relationship; Innovation and flexibility in ACP documentation processes
Hopkins 2020 [136]	UK	■	Barriers: Social distancing measures and visitation restrictions; Technological/telehealth barriers Facilitators: Triggers to initiate conversations
Hughes 2021 [137]	US	■	Barriers: Distrust in the healthcare system; Lack of awareness/knowledge of ACP Facilitators: Resources for clinicians; Language support services; Improved messaging
Janwadkar 2020 [138]	US	■	Barriers: Limited resources; Time constraints; Rapid disease progression; Social distancing measures and visitation restrictions; Technological/telehealth barriers Facilitators: Telehealth/virtual ACP platforms; Innovation and flexibility in ACP documentation processes
Kuzuya 2020 [139]	JP	■	Barriers: Social distancing measures and visitation restrictions; Rapid disease progression; Communication difficulties Facilitators: Telehealth/virtual ACP platforms; Guidance and protocols for ACP discussions; Information sharing; Care team collaboration
Moorman 2021 [140]	US	■	Barriers: Emotional barriers; Social distancing measures and visitation restrictions; Familial disagreement; Rapid disease progression; Financial concerns; Low education level; Racial and ethnic barriers; Strained healthcare system Facilitators: Trusting clinical relationship
Palipane 2021 [141]	UK	■	Barriers: Rapid disease progression Facilitators: Public awareness of ACP; Guidance and protocols for ACP discussions
Parekh de Campos 2021 [142]	US	■	Barriers: Limited resources; Time constraints; Lack of adequate ACP training for clinicians; Cultural and religious beliefs
Parks 2021 [143]	US	■	Barriers: Social distancing measures and visitation restrictions; Communication difficulties
Powell 2021 [144]	US	■	Barriers: Social distancing measures and visitation restrictions Facilitators: Guidance and protocols for ACP discussions; ACP/palliative care experts
Sinclair 2020 [145]	AU	■	Barriers: Personal protective equipment requirements Facilitators: Resources/education for patients/families; Telehealth/virtual ACP platforms; Resources for clinicians; Innovation and flexibility in ACP documentation processes
Swinford 2020 [146]	US	■	Facilitators: Telehealth/virtual ACP platforms; Care team collaboration; Guidance and protocols for ACP discussions
Van Buren 2021 [147]	US	■	Barriers: Family clustering of COVID-19; Social distancing measures and visitation restrictions Facilitators: Telehealth/virtual ACP platforms; Innovation and flexibility in ACP documentation processes; Care team collaboration; ACP/palliative care experts; Healthcare system improvements; Guidance and protocols for ACP discussions
Wallace 2020 [148]	US	■	Barriers: Rapid disease progression Facilitators: Guidance and protocols for ACP discussions; Resources for clinicians; Innovation and flexibility in ACP documentation processes; ACP/palliative care experts; Telehealth/virtual ACP platforms
Zaurova 2020 [149]	US	■	Barriers: Strained healthcare system; Rapid disease progression; Uncertainty surrounding the COVID-19 prognosis Facilitators: Early ACP; Guidance and protocols for ACP discussions; Triggers to initiate conversations

Notes: Abbreviations. Country: US = The United States; NL = The Netherlands; UK = United Kingdom; CN = Canada; IT = Italy; JP = Japan. Quality Assessment (level of evidence): ■ = Low; ■ = Low-Medium; ■ = High-Medium; ■ = High.

The COVID-19 pandemic spurred many experts in the field to write recommendations regarding ACP, emphasizing its importance and utility for healthcare systems [142,146]. The manuscripts within the Recommendation category emphasized the need for ACP to be patient- and family-centered, addressing both general and acute serious illness-specific preferences [124,125,126]. The authors of the papers within this category also suggested the necessity of innovations in ACP, including, again, the use of telehealth to facilitate discussions [126,127,128,130,145,146,147,148], and temporary policy changes or innovations to ease the completion and accessibility of ACP documents [135,138,145,146,147,148]. However, the urgency of the situation also highlighted the potential for misunderstandings and mistrust [125,137], suggesting the importance of clear, empathetic communication and ongoing dialogue [133,141,149].

There were recommendations that ACP discussions during the pandemic should encompass preferences concerning COVID-19 treatment options, ventilator use, and hospitalization [132,133,135,144,149]. The pandemic has prompted a reevaluation of ACP processes, encouraging a more nuanced approach that considers individual relationships, cultural, and religious aspects, within the evolving nature of the healthcare crisis [136,137,140,141,148]. ACP was recognized as useful for managing healthcare surges, respecting human rights, and enabling proactive care coordination [138,139]. A number of commentators suggested that the emphasis on ACP was expected to continue beyond the pandemic, with a push for incorporating these discussions as a routine element of care for all patients, particularly older adults and those with multiple health conditions [109,110]. Health and care organizations focused on older adults are encouraged to ensure up-to-date ACP policies, establish effective storage systems for ACP documentation, and provide comprehensive support to facilitate these critical discussions [144,145].

### 3.3. ACP Barriers and Facilitators Coding

The extracted ACP barriers and facilitators were codified and quantified; 165 barriers, categorized into 25 codes, and 250 facilitators, categorized into 21 codes, were identified. Table 6 shows the list of codified and quantified barriers to ACP and their frequency across the four categories. The most frequently occurring ACP barrier codes were: Social distancing measures and visitation restrictions (*n* = 35, 21%), Uncertainty surrounding the COVID-19 prognosis (*n* = 12, 7.1%), Technological/Telehealth barriers (*n* = 10, 5.9%), Lack of awareness/knowledge of ACP (*n* = 9, 5.3%), Limited resources (*n* = 9, 5.3%), Personal protective equipment requirements (*n* = 9, 5.3%), Rapid disease progression (*n* = 9, 5.3%), Strained healthcare system (*n* = 9, 5.3%), Time constraints (*n* = 9, 5.3%), Cultural and religious beliefs (*n* = 7, 4.1%), Discomfort among clinicians and patients discussing end-of-life care (*n* = 7, 4.1%), and Healthcare system barriers (*n* = 7, 4.1%).

Table 7 shows the list of codified and quantified facilitators to ACP and the frequency across the four categories. The most frequently occurring ACP facilitator codes were: Telehealth/virtual ACP platforms (*n* = 41, 16.4%), Training for clinicians (*n* = 29, 11.6%), Care team collaboration (*n* = 24, 9.6%), Innovation and flexibility and in ACP documentation processes (*n* = 22, 8.8%), Guidance and protocols for ACP discussions (*n* = 21, 8.4%), ACP/palliative care experts (*n* = 18, 7.2%), Resources/education for patients/families (*n* = 16, 6.4%), Identification of those in need of ACP (*n* = 12, 4.8%), Healthcare system improvements (*n* = 11, 4.4%), Improved messaging (*n* = 11, 4.4%), Resources for clinicians (*n* = 11, 4.4%), and Public awareness of ACP (*n* = 8, 3.2%).

**Figure 2:**
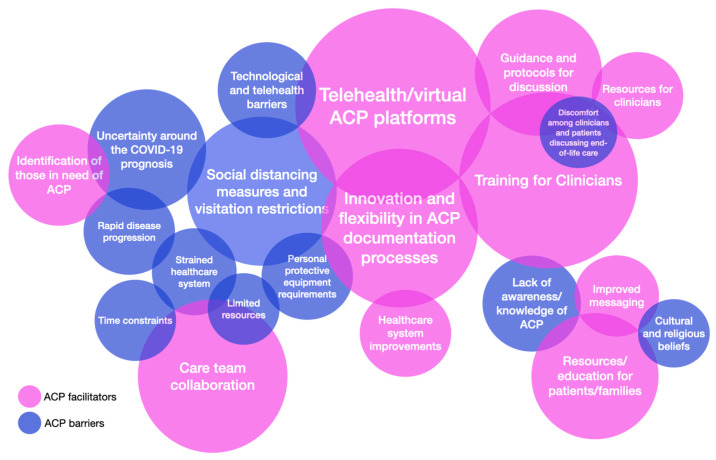
Bubble diagram illustrating the interaction of the major identified ACP barriers and facilitators during the COVID-19 pandemic.

Figure 2 shows a visualization in the form of a bubble diagram, of the identified ACP barriers (*n* = ≥7) and facilitators (*n* = ≥11) during the COVID-19 pandemic. The bubble diagram was created manually with the size and placement of the bubbles, respectively, representing the frequencies and interactions between the different barriers and facilitators.

### 3.4. Sub-Analysis of ACP Documentation/Engagement Increase and Decrease

Of the 115 included studies, 3 (2.6%) and 29 (25.2%) studies attested to decreased and increased ACP engagement/documentation during the COVID-19 pandemic, respectively. One study from Taiwan reported a 48% (1.9-fold) decrease in ACP engagement/documentation caused by the pandemic. The level of increase (% increase) in engagement/documentation ranged between 25.4% and 396% (mean 137.75%) according to the 18 studies that included analyzable data (Table 8). One of these studies also reports a 3163% increase in life-sustaining treatment (LST) documentation. Of the 34 studies that reported increased ACP, those that described ACP-related educational interventions and innovations (6) reported an increase in ACP of 25.4–101.6% (mean 55.2%). Studies that described ACP-related innovations (10) reported an increase in ACP of 33.3–396% (mean 151%).

## 4. Discussion

The purpose of this systematic review was to gain granular insights into the facilitators of and barriers to ACP during the COVID-19 pandemic. Analysis of the 115 included studies revealed 25 barrier codes and 21 facilitator codes, some of which were typical to ACP and others that were unique to, or amplified by, the pandemic situation. The findings of this study are in accordance with our previously published umbrella review, which aimed to identify the barriers to and facilitators of ACP implementation for healthcare workers across different settings [30]. In that study, we found frequent barriers to implementing ACP were visitation restrictions, a scarcity of resources and staff, poor collaboration among healthcare workers, an insufficient number of palliative care doctors, and the emotional strain on staff. The widespread uptake of telemedicine for communication was found to be the key facilitator of ACP. Other facilitating factors consisted of ACP/palliative care training, the integration of palliative care physicians into the acute care teams, and emotional support for medical staff [30]. The current study, while undergirding the findings of our previous study, builds upon that work by offering a deeper level of granularity. There follows separate discussions of the key barriers and facilitators identified in this study.

### 4.1. Barriers to ACP during the COVID-19 Pandemic

The findings of our review demonstrate that the COVID-19 pandemic has presented multifaceted barriers to effective ACP, necessitating a comprehensive understanding of these challenges to improve future practices. The most important and obvious barrier identified was the requirement for social distancing and restrictions on visitation in healthcare settings [36,37,39,40,42]. These measures, while deemed essential for infection control, intentionally isolated vulnerable populations and limited the face-to-face interactions with family members and healthcare professionals that are crucial for meaningful ACP discussions and shared decision-making. While telehealth emerged as an alternative to in-person consultations, it presented its own set of challenges [43,49,61,63,76]. The digital divide, particularly among older adults and those from lower socioeconomic backgrounds, has the potential to hinder access to telehealth services. Additionally, the nuances of ACP conversations, which often rely on non-verbal cues and a personal touch, could be lost in virtual settings.

Similarly, the necessity for PPE created a further physical barrier between healthcare providers and patients [39,40,44,48,53]. This not only made communication more challenging but also added an impersonal element to clinical interactions, which, in the context of ACP, are typically highly sensitive and personal. Furthermore, ACP discussions are typically time intensive due to their complexity and sensitivity; thus, the high volume of patients and the acute nature of COVID-19 treatment left healthcare professionals with limited time, which was identified as a barrier to ACP in various studies [44,45,49,55,76]. Indeed, the lack of time, PPE, hospital beds, medical equipment, and personnel were symptomatic of a healthcare system at breaking point. The overwhelming number of COVID-19 patients stretched healthcare resources thin [90,98,126,128,138]. Limited resources, including staff time and attention, impeded the ability to conduct thorough ACP discussions. The pandemic put an unprecedented strain on healthcare systems globally. This strain on healthcare systems, which was also noted as a barrier to ACP [40,48,55,61,92], led to a focus on managing acute cases and emergencies, often at the expense of important care planning conversations.

The COVID-19 disease itself presented a number of barriers to ACP. The novel and unpredictable nature of COVID-19 made prognostication challenging, complicating ACP discussions [98,124,125,128,149]. Uncertainty about the disease’s progression, outcomes, and long-term effects made it difficult for both patients and healthcare providers to make informed decisions about future care preferences. In particular, the rapid disease progression of COVID-19 infection was identified as a barrier to ACP in a number of studies [38,48,60,138,139,140]. Healthcare providers often found themselves making critical decisions rapidly, with limited input from patients or their families, potentially compromising the principles of patient-centered care and informed consent. The urgent focus on acute care for COVID-19 patients also often overshadowed the need for ACP, leading to missed opportunities for proactive care planning, especially for high-risk populations. Family clustering of COVID-19 was also identified as a barrier to ACP in one study [147].

The pandemic exposed a significant gap in public and sometimes professional awareness and understanding of ACP. This lack of awareness or knowledge of ACP was identified as a major barrier [55,64,66,86,90,125]. Some studies noted a need for adequate ACP training for clinicians as a barrier [76,91,142]. Moreover, the findings of our review indicate that certain communities experienced or were vulnerable to a lack of engagement in ACP due to a combination of factors; disparities in health education [90], cultural and religious beliefs [57,86,90,142], socioeconomic challenges [57,140], a general distrust in the healthcare system [65,124,125,137], and racial or ethnic barriers [53,90,124,140] played significant roles. These factors were compounded by the pandemic’s exacerbation of healthcare access disparities. This situation highlighted the need for more tailored, inclusive, and accessible approaches to health education and care planning, especially in underserved communities.

### 4.2. Facilitators of ACP during the COVID-19 Pandemic

The COVID-19 pandemic, while presenting numerous challenges, has also acted as a catalyst for several facilitators that have enhanced the process of and access to ACP. These facilitators have played a pivotal role in adapting ACP to the unique demands of a global health crisis. The rapid expansion and adoption of telehealth services has been a significant facilitator for ACP, identified in a large number of studies [93,96,99,100,101,102,104]. Virtual platforms have provided a means for clinicians and patients to engage in ACP discussions safely, overcoming the barriers imposed by social distancing and visitation restrictions as discussed above. This technology has made ACP more accessible, particularly for vulnerable populations or those in remote areas. Acevedo Rodriguez and colleagues, for example, describe their intervention to increase ACP among a population of veterans diagnosed with COVID-19, through direct telephone calls, which led to a 37% increase in ACP documentation and a 3163% increase in life-sustaining treatment (LST) documentation [104]. The videoconferencing software Zoom (Zoom Video Communications Inc., San Jose, CA, USA, 2016), was mentioned in numerous papers as a means for facilitating ACP or related educational interventions [67,83,86,111].

Alongside the telehealth-related innovations that have facilitated ACP conversation, this review also revealed notable innovation and flexibility in ACP documentation accommodating the constraints of the pandemic [116,117,118,119,120,121,126,127,128,135]. These innovations, which have facilitated the completion and accessibility of ACP documents, include streamlining the ACP processes [127], modification of the electronic health records to facilitate ACP documentation [71], the use of user-friendly [83] and electronic ACP forms [116,121,126,145], COVID-19-specific ACP documentation [135], apps to capture electronic signatures [63], electronic means of signature witnessing [138], and drive-by document signing [63]. As an example of how ACP documentation innovations can increase ACP documentation, McFarlane and colleagues describe a 296% increase in usage of the NHS’s Coordinate My Care services (https://www.coordinatemycare.co.uk, accessed on 14 May 2023), which takes an innovative integrative approach to care planning (including an ACP component) [117].

As described above, the need for adequate ACP training for clinicians, identified as a barrier to ACP, was addressed in numerous studies (largely in the Education category) that aimed to encourage ACP engagement through the training of health professionals [70,71,72,73,74,75,76,77,78,79,80,81,82,83,86,88,89]. During the pandemic, enhanced training programs for healthcare professionals have been crucial in equipping them with the skills necessary for effective ACP conversations, including clinical communication skills training and best practice in telehealth/virtual ACP. Casey and colleagues, for example, described a 25.4% increase in emergency department-based ACP following implementation of a “rapid and simple” educational program for emergency physicians on ACP [71]. Alongside such training, access to clinician-facing ACP-related resources [72,74,78,88,103] was also a facilitator to ensure that clinicians can navigate the complex and sensitive nature of ACP discussions, even under the stressful pandemic conditions. Resources for physicians included websites for palliative care/ACP information, visual aids, video materials, telephone call-center support, ACP tip sheets, etc. Among the most important resources for clinicians, identified as a ACP facilitators, were guidance and protocols for ACP discussions [139,141,144,146,147,148,149], including established guides such as Vital Talk [139,148] and COVID-19-specific goals-of-care discussion guides [94,112]. The development of specific guidance and protocols for ACP during the pandemic provided clinicians with clear frameworks to help ensure that ACP discussions are consistent, comprehensive, and adapted to the COVID-19 context.

Similar to the education and resources offered to healthcare professionals, public-facing ACP education and resources for patients and family members were also identified as facilitators for ACP [64,65,66,69,72,80]. The increased availability of resources and education for patients and families has been vital in facilitating ACP. This includes information delivered through websites, videos, community-based education, etc., about the importance of ACP, how to approach ACP conversations, and the specifics of healthcare decision-making in the context of COVID-19. For example, one 2020 study by Auriemma and colleagues showed that their free online resource, OurCareWishes.org, designed to guide patients and families through ACP, saw a 396% increase in ACP documentation after the onset of the COVID-19 pandemic [123]. Volandes and colleagues, in their large-scale nonrandomized controlled trial, implemented an intervention using ACP video decision aids and clinician communication during the pandemic that was associated with higher rates of ACP documentation (32% overall increase), especially for Hispanic and African American patients [50]. Increasing the availability and quality public-facing ACP resources, thus, could be particularly useful for reaching underserved communities.

Raising public awareness of ACP acted as an important facilitator for ACP engagement [42,45,48,54,58]. Among the studies included in our review were accounts of various efforts and recommendations to promote the need to engage in ACP, including large-scale initiatives [69], mass dissemination of ACP-related materials [58], and increased media coverage [42,54] around the necessity for early ACP decision-making. Tied to the need for improved resources, improved messaging [66,88,91,125,137] was also noted as a facilitator for ACP. Among the strategies to improve messaging were connections with community religious leaders and community organizations [58,137], the use of short videos [88], and motivational stories [125]. Enhanced messaging strategies regarding ACP have the potential to improve public awareness of its importance, leading to greater engagement in ACP. Indeed, the pandemic itself seems to have increased public understanding of the importance of ACP; the widespread fear of COVID-19, or an understanding of its dangers, has served as a motivator for both individuals and families to engage in ACP, recognizing the potential for rapid health deterioration and the importance of having care preferences documented [42,46,50,54,116]. Brophy and colleagues, for example, found that “perceived susceptibility to COVID-19 was a statistically significant positive predictor of intention to share one’s own EOL wishes” [46]. Comparing pre- and post-pandemic numbers, Connellan and colleagues reported a 291.5% increase in do-not-attempt-cardiopulmonary-resuscitation (DNACPR) documentation in older hospitalized patients [50].

The pandemic has fostered a greater sense of the need for collaboration among healthcare teams, which was identified as a major facilitator of ACP [92,94,95,96,105,111]. This teamwork was leveraged to help facilitate comprehensive, patient-centered ACP during the pandemic, ensuring that various perspectives and expertise are considered in care planning. A study by Singh and colleagues, for example, showed how social work and care management intervention, by involving social workers within the care team, increased Medical Durable Power of Attorney (MDPOA) documentation by 12.7% compared to baseline averages [105]. The care team collaboration that emerged from our review included the establishing of new, or developing preexisting, networks of support and integrated working within and between care teams and services, multi-disciplinary/interdisciplinary team engagement [96], an Interdisciplinary Ethics Panel (IEP) approach to decision making [92], and teams of professionals focused on proactively providing and supporting ACP [87,88,94,95].

In the same vein, a number of studies highlighted the involvement of ACP and palliative care experts, such as geriatricians, nurse practitioners, trained ACP clinicians, specialist palliative care clinicians, and chaplains, as an important facilitator of ACP [124,134,144,147,148]. ACP and palliative care experts can help to ensure that ACP is conducted with a high level of expertise and sensitivity, especially in complex and difficult cases or in those with advanced illness, of which there were many during the pandemic. Effective strategies to identify patients who would most benefit from ACP were also observed to be key facilitators [82,87,94,98,100]. These strategies included intensive outreach efforts to identify vulnerable patients, targeted programs aimed at those without ACP documentation, palliative care consultations, and the use of palliative care screening tools. As alternatives to in-person consultations, these programs often utilized telehealth technology, while some opted for direct postal mail. This proactive approach helped to deliver ACP to those at high risk of COVID-19 hospitalization/complications or those with significant healthcare needs. Similar to the proactive identification of those in need of ACP, the pandemic has encouraged an appreciation of a more proactive initiation of ACP conversations, with healthcare providers beginning discussions earlier in the patient journey [51,56,113,115,132]. In summary, these facilitators, among others, have collectively contributed to a more robust and adaptable ACP process that could weather the storm of the COVID-19 pandemic. This period of crisis has brought about significant learning and adaptation, potentially shaping the future of ACP to be more resilient, patient-centered, and integrated into routine healthcare.

### 4.3. Limitations

There are a number of limitations to this review that should be considered. First, the large number of included studies, while allowing for a high-resolution picture of ACP practice during the pandemic, also raised a number of issues with regards to data extraction, analyses, and quality assessment. These steps, because they were so time-consuming, were primarily conducted by the first author (T.M.) with careful oversight from the other authors to help mitigate potential bias. Second, inter-rater reliability measures for the manuscript screening showed only moderate agreement between the two reviewers. This could be due to a number of factors, namely, language differences and vision for the review; however, the final inclusion of papers was reached after lengthy discussion and debate, thus the included papers represent an agreed-upon selection. Thirdly, the majority (84.2%) of the included studies came from North America (*n* = 76, 66%) and the UK (*n* = 21, 18.2%), which biases the findings of our review towards these regions and perhaps does not give as full a picture of global trends as we originally sought to achieve. However, the US, Canada, and the UK are multicultural societies with healthcare systems in place that cater for people of various cultures, ethnicities, nationalities, languages, and faith traditions, which perhaps offsets this particular bias somewhat.

## 5. Conclusions

In this systematic review, we sought to investigate how the COVID-19 pandemic had affected the practice of ACP, the obstacles it presented, and the trends that emerged to facilitate ACP practice at a time when the healthcare system was strained to breaking point. In the published literature, the COVID-19 pandemic seems to have had a positive effect on the uptake and acceptance of ACP, or, at very least, it has increased general awareness of the importance and utility of ACP. The pandemic presented severe barriers, to ACP, such as strict social distancing measures, uncertainty surrounding the COVID-19 prognosis, the rapid disease progression, PPE requirements, and scarce resources and time, as well as exacerbating typical barriers to ACP, such as emotional, cultural, religious, educational, and racial barriers. However, the published literature from the first two years of the pandemic revealed a situation in which healthcare providers rose to meet those multiform challenges by finding innovative solutions to facilitate ACP, with trends towards widespread adoption of telehealth and flexibility in ACP documentation processes, utilizing multidisciplinary care teams and ACP and palliative care experts, and providing ACP-related guidance and recommendations, resources, and education to clinicians, patients, and caregivers. In summary, the findings of our systematic review showed that, for many counties, the COVID-19 pandemic, despite presenting many barriers, has been an opportunity for promoting ACP amongst diverse populations. Studying how healthcare providers rose to meet the challenges of delivering and promoting ACP during this pandemic can give us important insights for dealing with possible future medical crises and also for improving ACP practice in healthcare practice moving forward.

## Figures and Tables

**Table 1:** Characteristics of the included studies (*n* = 115) regarding advance care planning during the COVID-19 pandemic.

Study Characteristics		*n* (%)
Type of study	Nonrandomized	34 (29.6)
	Letters/Opinions/Editorials	28 (24.3)
	Quantitative Descriptive	20 (14.4)
	Qualitative	19 (16.5)
	Reports	7 (6.1)
	Mixed methods	7 (6.1)
Country	United States	70 (60.9)
	United Kingdom	21 (18.8)
	Canada	6 (5.2)
	The Netherlands	5 (4.3)
	Other	13 (11.3)
Setting	Hospital	42 (36.5)
	Nursing Home	21 (18.3)
	Online/Telehealth	15 (13.0)
	Electronic data	9 (7.6)
	Academic	5 (4.3)
	Other	6 (5.2)
Participants	Patients/Nursing home residents	58 (50.4)
	Healthcare professionals	34 (29.6)
	General public	9 (7.6)
	Family/Family caregivers	6 (5.2)
	Medical students	3 (2.6)
	Veterans	2 (1.7)

**Table 6:** Codified and quantified ACP barriers during the COVID-19 pandemic.

ACP Barrier Codes	Citation	Category				Total
		SI	ED	IN	RE	*n* (%)
Social distancing measures and visitation restrictions	[36,37,39,40,42,44,48,49,53,55,56,60,61,63,64,67,92,100,108,110,112,113,124,127,128,131,134,135,136,138,139,140,143,144,147]	16	0	6	13	35 (20.7)
Uncertainty surrounding the COVID-19 prognosis	[36,39,40,45,48,49,53,98,124,125,128,149]	7	0	1	4	12 (7.1)
Technological/Telehealth barriers	[43,49,61,63,76,80,124,136,138]	4	3	0	3	10 (5.9)
Lack of awareness/knowledge of ACP	[35,55,64,66,86,90,125,128,137]	4	2	0	3	9 (5.3)
Limited resources	[36,55,90,98,126,128,138,142]	3	1	1	4	9 (5.3)
Personal protective equipment requirements	[39,40,44,48,53,61,126,135,145]	6	0	0	3	9 (5.3)
Rapid disease progression	[38,48,60,138,139,140,141,148,149]	3	0	0	6	9 (5.3)
Strained healthcare system	[40,48,55,61,92,126,130,140,149]	4	0	1	4	9 (5.3)
Time constraints	[38,44,45,49,55,76,126,138,142]	5	1	0	3	9 (5.3)
Cultural and religious beliefs	[57,86,90,142]	2	4	0	1	7 (4.1)
Discomfort among clinicians and patients discussing end-of-life care	[36,38,49,91,126]	4	2	0	1	7 (4.1)
Healthcare system barriers	[36,49,55,90,126]	3	1	0	3	7 (4.1)
Emotional barriers	[46,48,82,97,140]	3	0	1	1	5 (3.0)
Communication difficulties	[57,76,139,143]	1	1	0	2	4 (2.4)
Distrust in the healthcare system	[65,124,125,137]	1	0	0	3	4 (2.4)
Legal concerns	[36,55,127]	2	0	0	2	4 (2.4)
Racial and ethnic barriers	[53,90,124,140]	1	1	0	2	4 (2.4)
Familial disagreement	[38,128,140]	1	0	0	2	3 (1.8)
Lack of adequate ACP training for clinicians	[76,91,142]	0	2	0	1	3 (1.8)
Negative perceptions about advance care planning	[45,124,134]	1	0	0	2	3 (1.8)
Financial concerns	[57,140]	1	0	0	1	2 (1.2)
Low education level	[76,140]	0	1	0	1	2 (1.2)
Blanket approaches to ACP	[134]	0	0	0	1	1 (0.6)
Family clustering of COVID-19	[147]	0	0	0	1	1 (0.6)
Low health literacy	[90]	0	1	0	0	1 (0.6)
Total		72	20	10	67	169 (100)

Abbreviations. SI = Situation; ED = Education; IN = Innovation; RE = Recommendation.

**Table 7:** Codified and quantified ACP facilitators during the COVID-19 pandemic.

ACP Facilitator Codes	Citations	Category				Total
		SI	ED	IN	RE	*n* (%)
Telehealth/virtual ACP platforms	[36,37,42,45,48,49,51,55,60,67,69,84,88,93,96,99,100,101,102,104,108,111,113,116,120,122,126,127,128,129,130,131,133,135,138,139,145,146,147,148]	11	2	14	14	41 (16.4)
Training for clinicians	[45,47,62,67,70,71,72,73,74,75,76,77,78,79,80,81,82,83,86,88,89,94,97,101,108,116]	6	18	5	0	29 (11.6)
Care team collaboration	[45,48,51,59,68,78,87,88,92,94,95,96,105,111,116,126,127,130,133,139,146]	5	3	9	7	24 (9.6)
Innovation and flexibility in ACP documentation processes	[48,63,66,68,71,116,117,118,119,120,121,126,127,128,135,138,145,147,148]	5	1	6	10	22 (8.8)
Guidance and protocols for ACP discussions	[45,52,64,71,82,87,92,94,107,112,124,129,139,141,144,146,147,148,149]	3	3	4	11	21 (8.4)
ACP/palliative care experts	[47,48,51,59,60,67,73,97,99,106,109,116,124,134,144,147,148]	7	1	5	5	18 (7.2)
Resources/education for patients/families	[36,54,64,65,66,69,72,80,83,85,97,121,123,127,145]	7	4	3	2	16 (6.4)
Identification of those in need of ACP	[36,40,53,82,87,94,98,100,106,110,124,133]	3	2	5	2	12 (4.8)
Healthcare system improvements	[49,59,68,94,105,108,116,129,133,147]	3	0	5	3	11 (4.4)
Improved messaging	[58,66,88,91,125,137]	2	2	0	7	11 (4.4)
Resources for clinicians	[54,62,72,74,78,88,103,109,137,145,148]	2	4	2	3	11 (4.4)
Public awareness of ACP	[42,45,48,54,58,69,97,141]	6	0	1	1	8 (3.2)
Proactive ACP conversations	[36,51,56,113,115,132]	3	0	2	1	6 (2.4)
Understanding/fear of COVID-19	[42,46,50,54,116]	4	0	2	0	6 (2.4)
Trusting clinical relationship	[49,84,135,140]	1	1	0	2	4 (1.6)
Early ACP	[135,149]	0	0	0	2	2 (0.8)
Language support services	[108,137]	0	0	1	1	2 (0.8)
Tablet computers	[60,108]	1	0	1	0	2 (0.8)
Triggers to initiate conversations	[136,149]	0	0	0	2	2 (0.8)
Individualized ACP discussion	[134]	0	0	0	1	1 (0.4)
Information sharing	[139]	0	0	0	1	1 (0.4)
Total		69	41	65	77	252 (100)

Abbreviations. SI = Situation; ED = Education; IN = Innovation; RE = Recommendation.

**Table 8:** ACP engagement/documentation increase during the COVID-19 pandemic (*n* = 18).

Study ID	Title	Country	Category	ACP Increase (%)
Auriemma 2020 [123]	Completion of Advance Directives and Documented Care Preferences During the Coronavirus Disease 2019 (COVID-19) Pandemic	US	IN	396
Copley 2021 [54]	ReSPECT (Recommended Summary Plan for Emergency Care and Treatment) in a Pandemic: The impact of COVID-19 on Advance Care Planning in a UK University Hospital Cardiology Department	UK	SI	355
McFarlane 2022 [117]	Advance Care Plans: Creation, Content and Use During Wave 1 of the COVID-19 Pandemic. A Retrospective Cohort Study of Coordinate My Care, a Large Electronic Palliative Care Coordination System	UK	IN	296
Connellan 2021 [50]	Documentation of Do-Not-Attempt-Cardiopulmonary-Resuscitation orders amid the COVID-19 pandemic	IR	SI	291.5
Hurlow 2021 [119]	An evaluation of advance care planning during the COVID-19 pandemic: a retrospective review of patient involvement in decision making using routinely collected data from digital ReSPECT records	UK	IN	230.9
Portz 2020 [121]	Advance Care Planning Among Users of a Patient Portal During the COVID-19 Pandemic: Retrospective Observational Study	US	IN	148
Yourman 2022 [101]	Acceptability and Effectiveness of Virtual Group Visits for Advance Care Planning	US	IN	Virtual ACP: 35 Clinic ACP: 136
Gaur 2020 [112]	A Structured Tool for Communication and Care Planning in the Era of the COVID-19 Pandemic	US	IN	114
Casey 2022 [71]	Advance Care Planning for Emergency Department Patients With COVID-19 Infection: An Assessment of a Physician Training Program	US	ED	101.6
Price 2021 [77]	An Educational Intervention to Increase Advance Care Planning Activities among Emergency Medicine Providers during the COVID-19 Pandemic	US	ED	98.4
Meyers 2022 [100]	Advance Care Planning in a Geriatric Veterans Primary Care Clinic During COVID-19	US	IN	78
Berning 2021 [87]	An advance care planning long-term care initiative in response to COVID-19	US	ED	46
Acevedo Rodriguez 2021 [104]	Improving the quality of care in patients with COVID-19	US	IN	AD: 37 LST notes: 3163
Hui 2022 [94]	Impact of an interdisciplinary goals of care program on hospital outcomes at a comprehensive cancer center during the COVID-19 pandemic: A propensity score analysis.	US	IN	33.3
Volandes 2022 [83]	Association of an Advance Care Planning Video and Communication Intervention With Documentation of Advance Care Planning Among Older Adults: A Nonrandomized Controlled Trial	US	ED	32
Rosedale 2022 [73]	Advance Care Planning and Health Equity: Pursuing Quality Improvement in a Family Medicine Residency Clinic	US	ED	27.9
Markwalter 2022 [79]	Advance Care Planning for Emergency Department Patients with COVID-19 Infection: An Assessment of a Physician Training Program	US	ED	25.4
Singh 2021 [105]	Increasing Medical Power of Attorney Completion for Hospitalized Patients During the COVID Pandemic: A Social Work Led Quality Improvement Intervention	US	IN	12.7

Abbreviations. Country: US = The United States; UK = United Kingdom; IR: Ireland, Category: SI = Situation; ED = Education; IN = Innovation; RE = Recommendation; LST = life-sustaining treatment.

## Data Availability

The original contributions presented in the study are included in the article, further inquiries can be directed to the corresponding author.

## Supplementary Materials

The following supporting information can be downloaded at: https://www.mdpi.com/article/10.3390/healthcare12060667/s1, Table S1: ACP barriers and facilitators during the COVID-19 pandemic—Situation Category: Extracted Data; Table S2: ACP barriers and facilitators during the COVID-19 pandemic—Education Category: Extracted Data; Table S3: ACP barriers and facilitators during the COVID-19 pandemic—Innovation Category: Extracted Data; Table S4: ACP barriers and facilitators during the COVID-19 pandemic—Recommendation Category: Extracted Data.
